# Effect of personal activity intelligence (PAI) monitoring in the maintenance phase of cardiac rehabilitation: a mixed methods evaluation

**DOI:** 10.1186/s13102-021-00350-9

**Published:** 2021-10-10

**Authors:** Amanda L. Hannan, Wayne Hing, Jeff S. Coombes, Suzanne Gough, Mike Climstein, Geoff Adsett, Rohan Jayasinghe, James Furness

**Affiliations:** 1 Faculty of Health Sciences and Medicine, Bond University, Gold Coast, QLD Australia; 2 Centre for Research on Exercise, Physical Activity and Health, School of Human Movement and Nutrition Sciences, The University of Queensland, Brisbane, Australia; 3 Clinical Exercise Physiology, School of Health and Human Sciences, Southern Cross University, Bilinga, QLD Australia; 4 Physical Activity, Lifestyle, Ageing and Wellbeing Faculty Research Group, University of Sydney, Sydney, NSW Australia; 5 Water Based Research Unit, Bond University, Gold Coast, Australia; 6 Cardiac Dynamics, Gold Coast, QLD Australia; 7 Cardiology Department, Gold Coast University Hospital, Queensland, Griffith University, Brisbane, QLD Australia; 8 Macquarie University, Sydney, NSW Australia; 9 Bond Institute of Health and Sport, 2 Promethean Way, Robina, QLD 4226 Australia

**Keywords:** Personal activity intelligence, Physical activity maintenance, Motivation, Barriers, Exercise therapy, Exercise, Fitness, Wearable

## Abstract

**Background:**

Personal activity intelligence (PAI) is a single physical activity metric based upon heart rate responses to physical activity. Maintaining 100 PAI/week is associated with a 25% risk reduction in cardiovascular disease mortality and 50 PAI/week provides 60% of the benefits. The effect of utilising this metric within a cardiac population has not been previously investigated. The aim of this study was to determine the effect of PAI monitoring on the amount and/or intensity of physical activity for people in the maintenance phase of cardiac rehabilitation and to explore participants’ perceptions of this approach.

**Methods:**

A concurrent mixed methods approach was undertaken. Participants in the maintenance phase of cardiac rehabilitation monitored PAI for six weeks via a wearable physical activity monitoring device (WPAM). In the first three weeks participants were blinded to their PAI score. A quality-of-life questionnaire (EQ-5D-5L) was completed, and semi-structured interviews conducted to investigate attitudes to PAI monitoring. Daily PAI data was collected throughout the 6-week period.

**Results:**

Twenty participants completed the trial. PAI earned/day was increased after participants could view their data (mean difference: 2.1 PAI/day (95% CI 0.3, 4.0), *p* = 0.027). The median change in percentage of days participants achieved a Total PAI score of 25 (*p* = 0.023) and 50 (*p* = 0.015) were also increased. The mean change in total scores for the EQ-5D-5L and EQVAS were improved after 6 weeks (0.6 ± 1.05; 95% CI (0.11–1.09); *p* = 0.019); (5.8/100; 95% CI (2.4–9.2); *p* = 0.002 respectively). Thematic framework analysis identified three global themes (perceptions on the WPAM, PAI and factors affecting exercise). Most participants stated motivation to exercise increased after they could view their PAI data. Many of the participants believed they would continue to use PAI long-term. Others were undecided; the latter primarily due to technical issues and/or preferring devices with greater functionality and attractiveness. All participants would recommend PAI.

**Conclusion:**

This exploratory study showed monitoring PAI via a WPAM increased the amount and/or intensity of physical activity within the cardiac population. Participants found PAI interesting, beneficial, and motivating. If technical issues, aesthetics, and functionality of the WPAM were improved, participants may continue to use the approach long-term. PAI may be a viable strategy to assist people with cardiac disease maintain physical activity adherence.

**Supplementary Information:**

The online version contains supplementary material available at 10.1186/s13102-021-00350-9.

## Background

According to the World Health Organisation (2019) cardiovascular disease (CVD) is the leading cause of death worldwide resulting in 17.9 million deaths per year; of which 85% were myocardial infarctions and strokes. A retrospective cohort study by Jernberg et al. [22] reported a 18.3% increase risk of future cardiac events within the first year, and 20% increased risk within the subsequent three years.

Coronary heart disease (CHD) is a large contributor to health costs. National Heart Foundation of Australia [34] estimated the cost associated with acute coronary syndrome to be $1,930.2 million in 2017–2018. Although CVD mortality appears to be decreasing in developed countries, the global aging population, growth, and longer-term survival rates from acute myocardial infarction is increasing the global economic burden. Deaths from CHD have been predicted to remain high into the next decade [22], [23].

Cardiac rehabilitation (CR) is an important secondary prevention strategy which comprises supervised exercise, education, and lifestyle modification. This health professional input is generally focused within the first three months post event, despite the risk of reinfarction remaining for several years [22]. Cardiac rehabilitation is described as having three phases. Phase one (Inpatient), Phase Two (6–12 weeks post event; subacute outpatient) and maintenance phase (period after the subacute recovery time) [34].

Adhering to life-long physical activity and maintaining a high level of cardiorespiratory fitness (CRF) is of paramount importance for people with cardiac disease, as CRF has been shown to be inversely proportionate to all-cause, and CVD mortality [24, 30, 32, 48]. Furthermore, the intensity of exercise is critical in reducing the risk of all-cause mortality, with more frequent, intense activity providing superior protection [19, 31, 32].

There is minimal research investigating the amount and intensity levels of physical activity completed by patients after the sub-acute phase of CR. However, from the limited available data, it appears few people who have been diagnosed with acute coronary syndrome continue to meet aerobic exercise guidelines long-term. A study by Kronish et al. [26] found that at five weeks post discharge, only 16% of participants were meeting the exercise guidelines recommended for those at two weeks post discharge. Further, a study by Reid et al. [44] found participants did not maintain increased exercise levels beyond two months’ post discharge from Phase 2. This suggests CRF improvements gained during CR are not likely to be maintained in the longer term and, therefore, the benefits of increased survival from higher CRF levels are not being realised in the cardiac population.

Lack of time and poor motivation have been identified in the literature as the most common barriers to performing regular physical activity in both healthy and cardiac populations [1, 3]. In recent years, wearable physical activity monitors (WPAM) have been introduced to address these barriers [12, 13, 16, 21]. A systematic review and meta-analysis by Brickwood et al. [4] reported consumer based wearable activity devices significantly increased step count, moderate and vigorous intensity exercise, and energy expenditure. Therefore, WPAM may be an answer to the call out for innovative solutions to assist in increasing physical activity levels across the lifespan as recommended by Peterman and Bassett [40].

A systematic review by Hannan et al. found that the use of WPAM in people with cardiac disease leads to a greater improvement in CRF, when coupled with exercise prescription or advice [18]. Mainstream exercise advice to walk 10,000 steps a day, or guidelines based on time alone (physical activity guidelines recommend 150–300 min of moderate intensity aerobic physical activity per week or 75–150 min of vigorous intensity aerobic physical activity per week [5]), do not emphasise the superior benefits in improving CRF gained by engaging in higher intensity activity and thus may be hindering cardiac patients’ ability to achieve optimal cardio-protection. While the main demographic of cardiac conditions is middle-aged or older, research shows older adults are receptive to, accept and can easily master WPAM [28, 29, 39].

While there are numerous activity trackers available, very few allow cardiac patients to track their activity and link this to useful metrics which reduce cardiovascular risk. Personal Activity Intelligence (PAI) tracks the amount of physical activity required to prevent CVD [37]. The PAI approach provides a simple metric providing feedback to users about whether the physical activity being performed is optimal to produce a reduction in risk for CVD, both in the apparently healthy population and for those with known CVD [25, 37].

The accumulation of 100 PAI/week is associated with a 25% reduced risk of mortality in healthy adults and 36% in patients with CVD (p < 0.001), regardless of whether traditional exercise guidelines were met [25]. Measuring PAI may address the common barriers to long-term exercise adherence by reducing the amount of time per week required to reduce CVD risk and encouraging higher intensity exercise to improve CRF. Currently, there appears to be no published studies evaluating the above hypothesis in a cardiac population, nor whether patients with cardiac disease would embrace a WPAM to allow for PAI monitoring.

We aimed to firstly determine whether monitoring PAI would influence the amount and/or intensity of physical activity performed by people with cardiac disease in the maintenance phase of CR. Secondly, we aimed to explore perceptions about WPAM use, PAI, impact on motivation, barriers to exercise, and predictions of long-term use.

## Methods

The study protocol was approved by the University’s Human Research Ethics Committee (173657). Participants were recruited from August to November 2019 through contacting cardiology clinics, CR programmes, a newspaper advertisement to the general community, word of mouth and expressions of interest at a local cardiac centre.

### Inclusion criteria

Individuals who were eligible for the maintenance phase of CR (≥ four weeks post-acute coronary syndrome, percutaneous coronary intervention, coronary artery bypass graft, and/or valvular surgery), with medical clearance from their treating cardiologist were eligible to participate in the study. In addition, participants were required be ≥ 18 and ≤ 80 years, own a smartphone with Bluetooth capability, be fluent in English to provide informed consent, be available to meet with the researchers on three separate occasions and be willing to wear a WPAM over the intervention period of six weeks.

### Exclusion criteria

Participants were excluded from the study if they were diagnosed with uncontrolled cardiac arrhythmias (particularly chronic atrial fibrillation), unstable angina, severe aortic stenosis, frequent premature ectopic beats, uncontrolled metabolic disease, chronic infectious disease, pregnancy, acute infection, undergoing chemotherapy or dialysis, uncontrolled positive exercise stress test, ejection fraction of < 40%, congestive heart failure, musculoskeletal, neurological, autoimmune disease or psychological issues impairing the ability to engage in physical activity.

### Study protocol

**Fig. 1:**
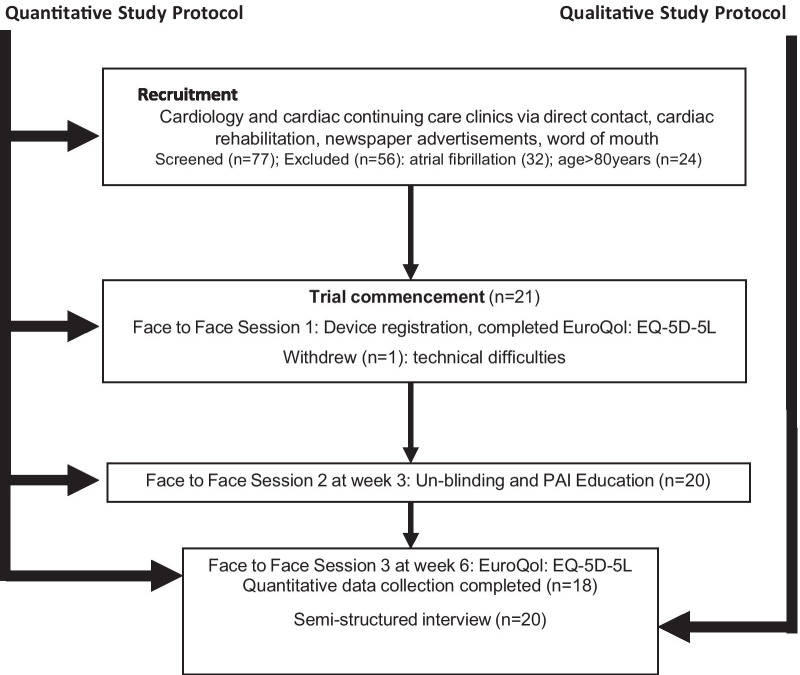
Concurrent mixed methods

A concurrent mixed method protocol was used incorporating purposive sampling. Figure 1 summaries the concurrent mixed methods protocol and methodologies used to address the research aims.

During the trial, researchers accessed the cloud based PAI Health research portal every two to three days to ensure data was uploading. Heart rates of participants were converted to a PAI score and displayed in the research portal. Participants who required a new charger or had technical difficulties with syncing the device were assisted to rectify the problem. No additional education or discussion about the trial occurred.

### Face to face sessions

Participants were required to meet the primary researcher, who is a physiotherapist and exercise physiologist, individually on three separate occasions (before intervention, at week 3 and 6) for thirty minutes each. Table 1 describes the procedures occurring at each session.

**Table 1:** Face to face session procedures

Session 1 individual face to face Before intervention	Session 2 face to face At 3 weeks	Session 3 face to face At 6 weeks
• Participant informed consent form completed and signed • Issued WPAM device • Register participant with PAI Health (age, sex, weight, height, resting heart rate, maximal heart rate calculated using formula from PAI Health App [211–0.64xage]) ** • Participants download the test-flight PAI research APP onto their smartphone which was used to blind subjects to the data • Instructed on daily syncing procedures, cleaning procedures (wiping to avoid grime and sweat interfering with accuracy of heart rate recording), charging procedures, and correct placement (2–3 fingers above the distal radius and ulna for the wristwatch: or two fingers below the deltoid muscle when using the arm strap) • Education on switching between all day and workout mode on device and to maintain usual activity level • Euro Quality of Life -5 Dimension-5 Level Health Questionnaire: English version for Australia (2009) completed **For participants on beta blockers or calcium channel blocker medication, 15 beats per minute was subtracted from the calculated maximum heart rate; or the maximum achieved heart rate on an exercise stress test was used	• Blinded App removed and un-blinded App installed on smartphone • Education about PAI given (verbal and written (handout) and three-minute video shown) • Explanations given that PAI is based on heart rate responses to exercise, and that higher intensity exercise would result in greater accumulation of PAI points • Educated on current research surrounding PAI, including the health benefits of maintaining 100 PAI • Participants who accrued under 10 PAI during blinding were encouraged to aim for 50 PAI initially to avoid unrealistic goal setting and disappointment in not achieving 100 PAI • Education about how to interpret the PAI APP, where to find the PAI score, seven-day bar graph, heart rate information and how to continue to sync the device was provided	• Euro Quality of Life -5 Dimension-5 Level Health Questionnaire: English version for Australia (2009) repeated • Audiotaped interview (either individually or in pairs) • Otter (speech to text software) used • Questions pertained to their experiences and opinions during the trial and projected thoughts for the future

WPAM, wearable physical activity monitor; PAI, personal activity intelligence; APP, application

### WPAM (wearable physical activity monitor)

The WPAM used for this study was a Lynk2 (NCI Technology, Inc. Oakbrook Terrace, Ill., USA). The Lynk2 uses photoplethysmography to continuously monitor heart rate with a display that uses a five colour LED light system which flashes and changes colour, and vibrates, as you move within heart rate intensity zones. It does not have a digital display. The device has a workout mode that allows more frequent sampling of heart rate data. Data (PAI, calories burned, training zones) is viewed on a smartphone/tablet using the PAI Health App. In this study we used the PAI Research App that has the same features as the commercially available PAI Health App however also allows for independent PAI data extraction from the PAI Health Database. During the blinded phase participants did not have access to data from the App. The data was collected from information synced from the WPAM. Participants could choose the location, time, and amount of exercise.

### Quantitative data analysis

Nes et al. [37] derived and validated a single physical activity metric (PAI) using the HUNT Fitness Study and general HUNT population which was associated with a reduced risk of cardiovascular disease mortality. Several studies utilising the PAI algorithm have been conducted across China and America [25, 35–37, 51]. Recently, a randomised controlled trial was implemented which monitored PAI in people with diabetes [9].

The PAI metric uses the heart rate response to exercise and is derived using a proprietary algorithm based upon individual heart rate reserve calculations over a seven-day rolling period. These measures are translated into an accumulated PAI score which is individually calculated based upon age, sex, resting heart rate, and maximal predicted heart rate, therefore allowing individual responses to physical exertion to influence scores. In addition, the algorithm uses calculations to ensure no more than 75 PAI can be accumulated in any one day and to make PAI harder to accrue after 50 PAI.

Daily PAI data was collected throughout the six-week period.

Table 2 illustrates the main PAI outcome measures. Total PAI scores were calculated using week 2 and 3 (blinded) and week 5 and 6 (un-blinded). This is because the first week was needed to generate a weekly PAI score. PAI earned/day was calculated for the two three-week periods (before and after un-blinding).

**Table 2:** PAI metric explanation

PAI metric	Explanation	Screenshot examples	
		A	B
Total PAI	Measured from the PAI earned during the last seven days. In the screenshot example A this is 126		
PAI earned/day	The amount of PAI earned per day. In screenshot example B, 19 was gained on Sunday		
Days > 25/50/75/100 PAI (%)	The number of days the Total PAI score was > 25/50/75/100 as a % of the number of days data was collected. From screenshot example B, the following can be calculated: PAI > 25 on 7 days = 100% PAI > 50 on 6 days = 86% PAI > 75 on 4 days = 57% PAI > 100 on 3 days = 43%		

### Statistical analysis of quantitative data

Raw data was collected and exported to the research group from PAI Health via password protected zip files. This raw data was imported into Excel and IBM SPSS (version 26) for analyses. Normality of distribution was considered for all analyses. Descriptive statistics were reported as mean (SD) or median (IQR), depending on the distributions of continuous variables over the course of the assessment. A paired *t*-test was used to investigate differences between the un-blinded and blinded phases. A simple linear regression was performed to determine whether age, body mass index, sex, medication use, time (months) since event and baseline PAI levels assisted with changes in the results. Multiple regression was performed for time since event. Based on responses to questions in the survey and information gained from the semi-structured interviews, participants were categorised according to their levels of motivation, intention of future use, ease of registering, problems with charging, problems with syncing, use of features, perceived comfort, number of barriers identified or level of understanding of PAI. The Mann–Whitney *U* test or the Kruskal–Wallis were then used to test for differences between categories for Total PAI and PAI earned/day.

### Quality of life

The Euro Quality of Life -5 Dimension-5 Level (EQ-5D-5L) [20, 43] survey was completed immediately prior to and on completion of the trial. Approval for use for non-commercial purposes was granted by The EuroQol Group Association (Registration ID: 30435). This quality-of-life instrument has been found to be reliable and valid for use in the cardiac population [11]. The EQ-5D-5Lcomprises two sections. The first is a rating score from 1 to 5 on perceptions about mobility, personal care, usual activities, pain/discomfort, and anxiety/depression, with 1 indicating no limitations and 5 indicating inability to perform the activity or extreme pain, anxiety, and depression. The total score was out of 25. The second component (EQVAS) [20] involves participants rating themselves on a visual analogue scale. Participants rated their health on a scale of 1–100 (1 being the worst health they could imagine and 100 being the best health they can imagine).

### Qualitative data methods

Each participant who completed the trial undertook a semi-structured interview of approximately thirty minutes duration, comprising eighteen questions (Additional file 1). The Consolidated Criteria for Reporting Qualitative Studies (COREQ): a 32-item checklist adapted from Tong et al. [49] was utilised and presented as supplementary material (Additional file 2). The researcher conducting the interviews (AH) had previously interviewed seventeen healthy participants, in a preliminary pilot study. This study was used to test the logistical aspects of the current study. The participants in the pilot study underwent the identical 6-week methodology of PAI monitoring used in this study, along with identical questions in semi-structured interviews. The interviewer was a female Physiotherapy Lecturer at a University who had no previous relationship with any of the participants. This was all the information participants knew about the researcher. The interviewer has an interest in cardiovascular research. There was no other person present at the interviews, other than the researcher and participant.

The questions were written to explore the participants’ perceptions surrounding their experience of partaking in the trial and focused on whether the WPAM influenced their physical activity; particularly comparing the blinded period versus being able to visualise the PAI score. Participants were also asked questions to determine their understanding of the concept of PAI, to identify barriers to their exercise; both generally and during the trial period, whether they attended a CR programme and whether they felt they had been given adequate guidelines surrounding exercise after their cardiac event. Finally, the participants were asked whether they believed they would continue to use the device and/or PAI concept in the future as well as the likelihood they would recommend the concept to other people. Additional questioning was used to clarify answers if required, and to reach saturation, hence not all interviews were identical.

These semi-structured interviews were conducted using Otter software and downloaded to a word document. Recordings were replayed verbatim to check for accuracy and develop transcripts. Changes to the transcripts occurred to improve accuracy when software incorrectly recorded words or sentences. Transcripts were uploaded to NVIVO 12 software [42] for thematic analysis.

A systematic approach of thematic framework analysis as described by Ritchie and Spencer [45] was implemented to identify common themes. This approach consisted of five steps and were completed by two authors (AH and SG):

1. Familiarisation of data (notes were made when key ideas, thoughts or concepts were similar across participant transcripts)
2. Identifying a thematic framework (key ideas and priori issues were used to start the coding tree and further refinements made as additional similarities of themes emerged). A second researcher reviewed the data and a consensus on themes/subthemes were determined to reduce researcher bias and ensure a valid and reliable analysis was performed [2].
3. Indexing (within NVIVO 12, all textual data in individual transcripts were indexed to correspond with the global themes derived from the data)
4. Charting and mapping (data was extracted from the original transcripts and placed in a chart consisting of headings and subheadings that were derived during steps 1–3) and,
5. Interpretation (transcripts were mapped to identify further commonalities and interpretation of the data was presented).

Finally, the perceived effect of PAI on motivation, intention to use the device in the future, ease of use, comfort, usage of device features, understanding of PAI, attending CR, perception of receiving adequate exercise guidelines and number of barriers to exercise identified, were explored to ascertain whether these affected Total PAI and PAI earned/day.

## Results

### Demographics

21 participants (16 males; 5 females) were enrolled in the study. Three participants were recruited via a newspaper article, three via word of mouth and fifteen via a cardiac care centre. One 79-year-old female participant withdrew from the trial due to technical difficulties with syncing the device and time constraints. Another two participants’ (1 male and 1 female) data were excluded from the quantitative analysis due to a decreased opportunity to undertake physical activity in the un-blinded part of the trial. This was due to external factors (bushfire evacuation, moving overseas and sustaining an injury), which were out of their control. Table 3 depicts participant characteristics and breakdown of individual participant diagnoses.

**Table 3:** Participant baseline characteristics n = 18; (values are mean ± SD or number (%) or months since event)

Male sex, n (%)	15 (83)
Female sex, n (%)	3 (17)
Age, years	56 ± 15.5
Body mass index, kg/m^2^	26.5 ± 4.4
Cardiac history n,	(months since event)
Percutaneous intervention	6 (1,3 × 18,24,2 × 36)
Myocardial infarction	1 (6)
Myocardial infarction with stent insertion	3 (4,5,8)
Coronary artery bypass graft surgery	1 (24)
Myocardial infarction and coronary artery bypass graft surgery	1 (4)
Coronary artery bypass graft surgery with stent insertion	1 (5)
Valve surgery	3 (9,24,36)
Valve surgery with pacemaker	1 (10)
Myocardial infarction with stent and ICD insertion	1 (6)
Mean average time (months) from cardiac event	15.2 (12.1)
Medication affecting heart rate	
Beta blockers, n (%)	3 (17)
Calcium channel blockers, n (%)	2 (11)
Attended CR phase 2, n (%)	10 (56)
Perceived adequate exercise guidelines given post event, n (%)	6 (33)

SD, standard deviation, kg, kilograms, cm, centimetres, x, times

Of the twenty participants who were interviewed as part of the qualitative analysis, 60% attended a CR programme post cardiac event, 15% were offered admittance to CR, however subsequently declined and 25% of participants were never offered CR. Only 35% percent of participants believed they were given adequate exercise guidelines post event, 10% were unsure whether the guidelines given were appropriate and 55% of participants expressed they were not given adequate exercise guidelines post cardiac event.

Besides the three face to face sessions, additional assistance was required for 45% of participants. Of these, one participant required assistance six times due to inability to navigate the syncing (WPAM with smartphone app) process, one participant required assistance when the registration with PAI Health failed and on three occasions when technical difficulties arose with the device. A further three participants experienced failed registration and technical difficulties (one participant required changing of the device twice as the charging mechanism failed due to a faulty connection) and the remaining three participants had one episode each where technical issues required assistance. No additional advice or discussion around the study was given to participants during resolution of technical difficulties.

### PAI data

Table 4 provides the PAI data for the blinded and un-blinded phases. Both medium (*p* = 0.07) and high intensity mean differences (*p* = 0.15) increased after viewing PAI, although not to statistically significant levels. There were statistically significant (*p* < 0.05) increases in the percentage of days participants achieved 25 and 50 PAI.

**Table 4:** Personal activity intelligence (PAI) metrics during the blinded and un-blinded phases

	Blinded mean (SD)	Un-blinded mean (SD)	Mean difference (95% CI)	*p*-value
Total PAI	68.4 (65.2)	97.6 (73.5)	29.2 (7.2, 51.2)	0.012*
PAI earned/day	9.9 (8.47)	12.0 (7.49)	2.1 (0.3, 4.0)	0.027*
PAI in low intensity	1.5 (1.65)	1.4 (1.5)	-0.5 (-0.2,0.2)	0.71
PAI in medium intensity	5.4 (5.1)	6.4 (5.2)	1.0 (-0.3–2.1)	0.07
PAI in high intensity	3.1 (4.4)	4.2 (3.7	1.0 (0.4,3.0)	0.15

	Median (IQR)	Median (IQR)		*p*-value
Days > 25 PAI (%)	71.0 (5.77,100)	100 (73.5,100)		0.023*
Days > 50 PAI (%)	18.3 (0,100)	80.77 (28.9,100)		0.015*
Days > 75 PAI (%)	0 (0,100)	45.39 (0,100)		0.116
Days > 100 PAI (%)	0 (0, 94.65)	10 (0,100)		0.344

SD, standard deviation; CI, confidence interval; IQR, inter quartile range, *statistically significant *p* < 0.05

**Fig. 2:**
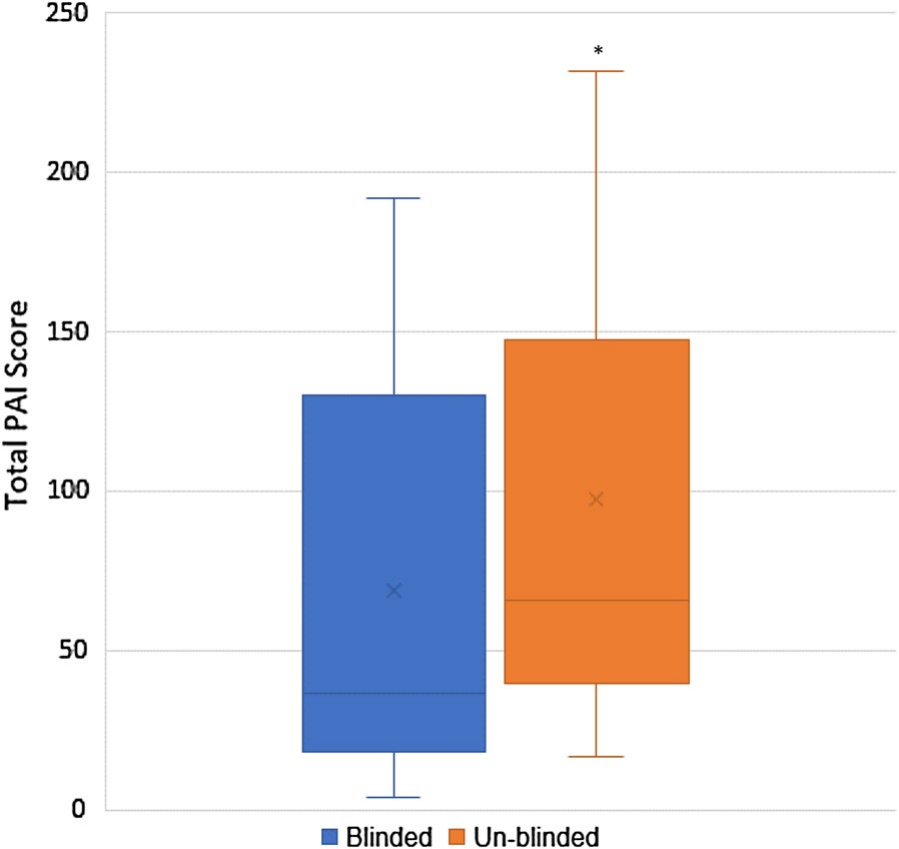
Mean total PAI score before and after un-blinding. Where: * = *p* ≤ 0.05. Explanation of box plot: the cross represents the mean. The vertical lines depict the minimum and maximum values. The horizontal line represents the median. The bottom line of the box depicts the median of the first quartile. The top line of the box depicts the median of the third quartile.

There was a significant increase in change in PAI earned/day and Total PAI once participants could view their data (Fig. 2).

**Fig. 3:**
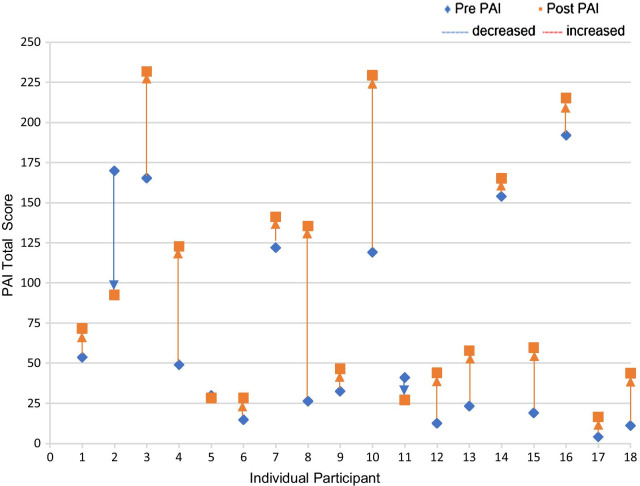
Individual participant changes in total PAI score before and after un-blinding

Figure 3 shows the individual participant changes in Total PAI Score before and after un-blinding. A total of 89% (16/18) of participants increased their Total PAI after being educated on PAI and able to view their PAI data. In the blinded period (3 weeks), six participants (33%) were achieving less than 25 PAI per week and this reduced to one participant (5%) at the completion of the trial. One participant (5%) was achieving between 50 and 75 PAI at three weeks and this increased to three (17%) at six weeks. The number of participants reaching 50 PAI or above increased from 7 (39%) (at 3 weeks) to 11 (61%) at six weeks with a further two participants achieving close to 50 PAI (46 and 44). For the six participants who were already achieving 100 PAI per week at baseline (3 weeks), half of them increased PAI score to over 200 PAI per week, two participants achieved > 230 PAI and one participant dropped below 100 PAI to 93 once data was un-blinded.

### Linear regression

Linear regression looked at associations between the change in PAI earned/day and change in Total PAI with age, sex, body mass index, medications affecting heart rate, time from cardiac event in months and PAI at baseline of each phase. Table 5 shows that time from cardiac event was significantly associated with the change in Total PAI (*p* = 0.008) This indicated that participants were more likely to increase their PAI if their cardiac event had occurred closer to the study. Multiple regression showed the association between time from cardiac event and change in Total PAI was independent of the other factors and for every month further from participants’ cardiac event, the change in Total PAI significantly decreased. by 2.2 (coefficient, 95% CI = -2.2, -3.8 to -0.5; *p* = *0.013*) (Table 6).

**Table 5:** Simple linear regression for change in PAI earned/day and change in total PAI

Factor	Change in PAI earned/day			Change in total PAI		
	Coefficient	95%CI	*p*-value	Coefficient	95%CI	*p*-value
Age, years	− 0.1	(− 0.2,0.1))	0.2	− 0.4	(− 1.9,1.1)	0.57
Sex (female)	0.8	(− 4.3,6)	0.74	3.7	(− 57.9,64.6)	0.91
Medication (no)	0.3	(− 4.3,4.9)	0.89	7	(− 47.8,61.7)	0.79
BMI kg/m^2^	− 0.3	(− 0.7,0.2)	0.21	− 2.4	(− 7.6,2.8)	0.34
Time from event, months	− 3.4	(− 6.8,0.1)	0.051	− 2.2	(− 3.8, − 0.6)	0.008*
Baseline PAI	0.2	(0, 0.4)	0.051	0.1	(− 0.3,0.5)	0.58

^*^Statistically significant *p* < 0.05

**Table 6:** Multiple regression results: change in total PAI

Factor	Coefficient	95%CI	*p*-value
Constant	1.8	(− 37.8,41)	0.93
Time event, months	− 2.2	(− 3.8, − 0.5)	0.013*
Baseline PAI total	0.0	(− 0.3, 0.3)	0.834

^*^Statistically significant *p* < 0.05

No statistically significant differences were found in the levels of the categorical variables tested: motivation (same/a little/a lot), intention to use in the future (no/yes/unsure), ease of registering (easy/some problems/difficult), charging (easy/some problems/difficult), syncing (easy/some problems/difficult), use of features (did not use/used), perceived comfort (no/yes), number of barriers identified (0/1/2/3/4/5) or level of understanding of PAI (fair/good).This may be due to the small sample size and no definitive conclusion can be made. There was also no difference found in either pre–Total PAI or baseline PAI earned/day for those attending CR versus not attending; nor was baseline Total PAI or baseline PAI earned/day different for those perceiving they received adequate exercise guidelines versus those who did not.

### Quality of life

Data from the EQ-5D-5L Health Questionnaire using a paired t-test indicated that there was a significant improvement in the total quality of life score from enrolment levels to six weeks (mean change 0.6 ± 1.05;95% CI (0.11–1.09); *p* = 0.019). The EQVAS score was also found to be significantly different from enrolment levels after 6 weeks (5.8/100; 95% CI (2.4–9.2); *p* = 0.002). There were no significant changes across the EQ-5D-5L domains of mobility, personal care, usual activities, pain/discomfort or anxiety/depression.

### Qualitative data results

**Fig. 4:**
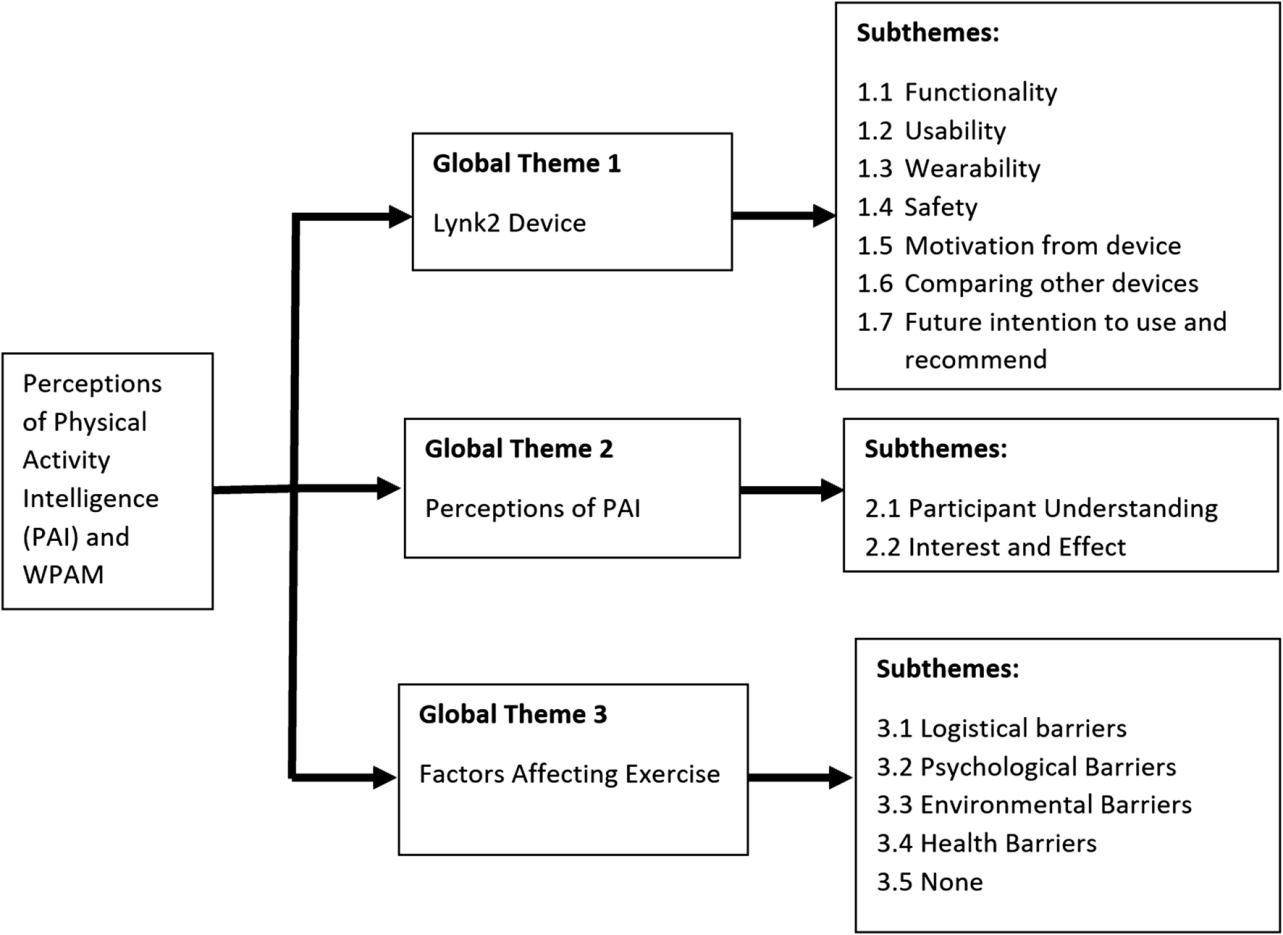
Diagrammatic representation of the themes and subthemes within the thematic framework

The thematic framework analyses identified three global themes derived from the initial coding tree which consisted of six nodes; Lynk 2 device, concept of PAI, barriers to exercise, cardiac rehabilitation, exercise guidelines post event and impact of blinded data. The global themes were further broken into fourteen subthemes as illustrated in Fig. 4. Table 7 represents each global theme, subthemes and presents examples from the participant transcripts for each subtheme.

**Table 7:** Themes, subthemes and transcript examples

Subtheme	Examples
Global theme 1 Lynk2 device	
1.1 Functionality	“In terms of being able to use the device itself, I found it really easy to use.” (P14)
1.2 Usability	“I noticed that quite often on the hill because I sort of, there were three ups and down, so on the down I could feel it vibrate coming down or up and yeah, I know I would press it to look at the colour and see how it's going.” (P15)
1.3 Wearability	“I didn't even know it was there at any time unless I was particularly attending to it.” (P10)
1.4 Safety	“It gets caught on your clothing". (P16)
1.5 Motivation from device	“Yes, well, I had a bit of a sticky start because I didn't know I wasn't well so but once I got over that, I found it motivating too. You want to see the mechanics of it working.” (P13)
1.5 Comparing other devices	“I like the idea of this, over a Fitbit because I've also had a Fitbit but the fact that when I was working with the Fitbit, I was doing 20,000 steps a day, but I mean I could do that just for a stroll.” (P15)
1.7 Future intention to use and recommend	“So, you're highly likely to continue to wear the Lynk2 device so could you talk to me about why you want to keep wearing it?” (Interviewer) “Just so I can monitor my training, it's actually giving me something; a goal to achieve. Yes, I found that quite, quite good, in the sense that I've been able to watch and monitor my training as I go during the day.” (P8)
Global theme two: perceptions of personal activity intelligence	
2.1 Participant understanding	“Well, I thought just walking or doing, you know, the 10,000 steps, just walking would be adequate. But, you know, I learned that through the PAI that it's not just exercise. It's exercise with increasing the heart rate that actually provides you with PAI. So that that was a real eye opener. So, walking all day at work, was not necessarily going to give me the PAI that I required.” (P18)
2.2 Interest and effect	“You can, I guess, be healthier and less chance of another heart attack and possibly more years to your life if you keep over that hundred, hundred PAI and although it probably didn't affect me as much as it's probably affected some of the others, that haven't done a lot of training. But some days where you think you’re going to do 30 PAI and you do 10 PAI, it was interesting to see some of the exercise sessions that I do that are really probably not working as hard as you think it might have been. Especially if I'm doing heavy weights. Yeah. So, it doesn't tend to tax the heart that much.” (P3)
Global theme 3 factors effecting exercise	
3.1 Environmental barriers	“a couple of times, it was just really hot and that affected not so much my capacity to exercise just my motivation to exercise.” (P7)
3.2 Logistical barriers	“It's hard like working in Brisbane. I get up at quarter past three and leave here at four o’clock and I don’t get home until night-time.” (P4)
3.3 Psychological barriers	“I find exercise boring.” (P17)
3.4 Health barriers	“Only barrier is, I have, is I can't run as I snapped my Achilles Tendon, so I need to do something like rowing or boxing, something that doesn't involve sprinting.” (P5)
3.5 No barriers	“No, no barriers at all.” (P19)

## Global theme 1 perceptions of wearable physical activity monitoring device (Lynk2)

### Functionality

The perception of the WPAM functionality pertained to ease of registering, charging, and syncing the device. These functions are mandatory requirements to allow ongoing use of the device.

#### Registering

With respect to registering, most participants found registering straight forward and easy to complete. Some participants had difficulty registering. There were difficulties with the PAI App at time of registration where the email address and internet connection caused registration to fail [“Well, initially, it was a few days of touch and go trying to get it to register.” (P1)]. One participant had an old phone whereby outdated software needed upgrading (“I just think it was the phone.” (P6)) and a further participant had problems with the App not coming up [“I did have a little bit of trouble initially. Sometimes, it wouldn't come up at all.” (P5)].

#### Charging

Charging the device was perceived as a positive experience for the majority of participants and one participant commented positively on the battery life [“... It's such a small item you could just click it beside your bed each night and, but the other thing I found which was really positive, was it lasted a long time. Wearing it full time, so that was, I was happy about that.” (P19)]. The main reasons identified by participants who found charging more difficult were remembering to charge the device, malfunctioning of the charger itself*,* and the charger playing up.

#### Syncing

Participants commented syncing the device was easy or they had no difficulties. However, some participants found it more challenging [“… it was messy and tricky for me. Sometimes I had to try ten times a day to sync.” (P18)]. Comments also included difficulty with it syncing when you wanted it to, internet issues and difficulties with the phone application.

### Useability

Useability pertained to understanding the features, and their usefulness. Features included the vibration and colour change to indicate change of intensity, workout mode, and the application on the phone, including the PAI score. Some participants reported not understanding all aspects of the features [“I have had some difficulties electronically because I keep forgetting what the flashes are for.” (P10)]. For those participants who did use the features, the vibration, colours, exercise/sport mode, bar graph and application/PAI were all found to be useful by different participants. However, a couple of participants found the features confusing [“That seems awfully strange because I've been sitting on the lounge, and it’s vibrated a couple of times … and I'm just sitting around watching TV.” (P11)]. Finally, several participants also commented they would have preferred the device if it had a watch component as a feature.

### Wearability

Wearability pertained to device comfort. The device was described as comfortable by most participants however there was mention of some unfavourable aspects. Favourable comments included not even noticing the device was on and having no problems with it. Less favourable comments included it was annoying, uncomfortable, hot, and interfered with their job. Finally, one participant was bothered that it did not match her jewellery [“I had jewellery on, and it just didn't quite go yeah, I had to ditch the Black, but I didn't want to ditch the device. So yeah, that's just me being honest and vain all at the same time.” (P14)].

### Safety

Safety concerns of the device included catching on clothing and a couple of participants developed skin irritation [“Might not do it to everybody else obviously but to me that gave me a bad rash about my shoulder.” (P15)].

### Motivation from device

Results revealed strong agreement from participants that they were motivated to perform more, or higher intensity exercise when they were able to view the PAI data, although some described no change in motivation. No participants reported having decreased motivation as a result of wearing the device, however one participant mentioned not attaining his PAI made his mental health worse [“I am a stressful type of person and when I see that go down it really stresses me out and I don't know, probably good for my health but not good for my mental stuff.” (P9)].

Reasons participants gave for their increased motivation from the device included that it helped them get started for the day, they wanted to increase PAI points, and it helped participants work harder. Participants who were already doing 100 PAI at baseline reported their motivation to exercise stayed the same, despite achieving higher PAI scores after viewing PAI data. Two participants decided to delay regular exercise until the New Year and identified motivation to exercise did not change once PAI data was made available [“I want to change myself after Christmas, when everything settles down.” (P11)].

### Comparing other devices

Numerous participants had never used any type of fitness device before the trial. Of those that did, there were varying views as to whether they preferred the Lynk2 device or another device they had used previously. Reasons given by those who preferred the Lynk2, were they felt it was more accurate and they liked the PAI score because it was more individualised and was based on intensity of exercise. Other participants preferred a Garmin® device, one mentioned they preferred a Polar and another a Fitbit or Apple watch. Reasons given were these devices offered further features such as telling time, notifying phone calls/texts and being more aesthetically pleasing.

### Future intention to use and recommendation

Participants’ views about whether they believe they would continue to use the device on an ongoing basis was variable. Some participants expressed strong indications they would continue to utilise the device for health benefits, motivation, and monitoring training. Other participants were hesitant to commit to continued use. For participants who were undecided, views were expressed that if the device also told time and the syncing improved, they would be more likely to utilise the device in the future. Finally, the remaining participants remarked they would not continue to wear the device after the trial as they found the device annoying or because it didn’t show data in real time on the device, only through the phone application.

Despite, varying opinions about future use, there was overwhelming agreement from participants that they would recommend the device to others. Reasons for recommendation included believing it was beneficial, liking the PAI concept, improving motivation to exercise and it is a great tool. Only one participant stated he would not recommend the device as he believed it did not have enough features compared to other devices; however, he also mentioned he would recommend the PAI concept:

"I don't know. the device, probably not. The PAI, yes, I would, but not the device. I get the PAI. I think the problem is these days people have these devices, and they do 1000 things. Someone wants something more that they can wear all day, and all monitor the heart, you know, most smartphones well they can do that these days. Yeah. And if I'm going to wear a device it's going to tell me about a text message, phone call, and it's going to probably track my sleep patterns and so forth. So probably not the device". (P9)

## Global theme 2 perceptions of personal activity intelligence (PAI)

### Participant understanding

Most participants reported that they understood the concept of PAI.

"After the three weeks where you can actually, when you understand what you’re, where you're heading with it all, and you understand what PAI means and you can actually see the numbers that, I think being a guy too, you kind of, maybe the competitiveness, you want to keep it over a certain number". (P3)

Other participants had difficulty understanding that to gain PAI, you had to do exercise that increased your heart rate [“one day, I thought I'd done quite well, and it came up with zero. Yeah. and I thought I did quite well and had pushed myself. I thought there must be something wrong with it. I’d done all that work and got zero.” (P6)].

### Interest and effect

All participants found the concept of PAI of interest, particularly mentioning the individualised nature of it and being able to monitor activity [“Okay, well, this is the first time I learned about PAI. I think it's a really interesting concept, and I like the fact that it's a personal thing as opposed to a whole population thing….” (P7)]. However, a couple of participants reported they only did the trial to help the researchers [“I just thought I'd have done the trial and that was it.” (P6)]. In addition, some participants commented they became more aware of the amount and intensity of the exercise they were performing and increased their intensity [“I'll take longer walks and I find hills, I found a hill to walk up and this morning I did it twice.” (P6)].

## Global theme 3 factors effecting exercise

### Logistical barriers

Participants reported logistical difficulties such as commuting to work; caring responsibilities, lack of time, and travel contributed most to a reduction in exercise over the trial period. As the trial for some participants ran over Christmas, additional shopping needs also competed with time to exercise. Finally, a couple of participants mentioned the logistics of having to remember to wear the device also contributed to a reduction in PAI.

### Psychological barriers

Psychological barriers were identified as lack of enjoyment of exercise, fear of having another cardiac event or exercising too hard, anxiety and mental stress.

### Environmental barriers

Participants identified environmental factors including outside temperature (heat was identified as contributing to fatigue), discomfort and demotivation, air pollution (smoke), bushfires and rain as being barriers to exercise participation during the trial.

### Health barriers

Health issues from illness and injury prior to and during the trial were reported as contributing to the type and amount of exercise performed. Previous injuries included shoulder dislocations, back, foot/ankles, Achilles’ tendon rupture, knee problems and periods of bad health. During the trial, three participants went to hospital due to previous health struggles, and for a pre-scheduled minor operation. Another participant had a skiing accident and was unable to exercise for two weeks. Additional health factors identified as affecting exercise included premenstrual issues, cold and flu, lack of energy, blood pressure issues and shin splints.

### No barriers

There were some participants who expressed having no barriers affecting exercise.

## Discussion

This is the first study to investigate the use of PAI in a cardiac population as an intervention. We aimed to determine whether monitoring PAI, would influence the amount and/or intensity of physical activity performed by people with cardiac disease in the maintenance phase of CR. Secondly, we aimed to explore perceptions about the ease of use of the WPAM, impact on motivation, barriers to exercise, and predictions of long-term use. The main finding from this exploratory study was that Total PAI and PAI earned/day increased after participants were able to see their PAI score and all participants would recommend PAI monitoring to others, suggesting it was effective and well received in the cardiac population.

Most participants who completed the trial were male (83%), which represents male dominated enrolment as typically seen in CR [46], however the trial under represented females. The breadth of participant diagnoses and cardiac interventions were also a good representation of the cardiac population [33].

Our results showed no significant difference in PAI scores between participants who did or did not attend Phase 2 CR nor between those who believed they were given adequate exercise guidelines post cardiac event or not. This is interesting as one would have thought with greater health professional input, Total PAI score and PAI earned/day may have been greater.

Overall, our analyses showed the Total PAI scores and PAI earned/day increased after participants were educated about PAI and were able to see their PAI values. This coupled with almost ninety percent of participants increasing their scores and all participants stating they would recommend PAI monitoring to others suggests it was effective and well received in the cardiac population. This was also supported by the qualitative results showing the majority of participants commented that their motivation to exercise increased when being able to view the data and they tended to exercise harder to try to accumulate more PAI points.

The accumulation of 100 PAI/week is associated with a 25% reduced risk of mortality in healthy adults and 36% in patients with CVD (p < 0.001) and resulted in five years longer lifespan [25, 37]. Additionally, 100 PAI/week reduces the risk of mortality by 30.5% for people who are overweight, 31.5% for those with hypertension, 54% with type 2 diabetes and 31.5% for those who smoke [25]. In addition, for those maintaining a Total PAI score of 50/week, around 60% of the health benefits are gained [25]. Our findings showed two thirds of participants (61%) reached 50 PAI/week or above once data was able to be viewed compared with 39% at three weeks. Furthermore, 89% of participants increased Total PAI/week to some extent, likely reducing CVD risk compared to pre-trial levels.

Our results indicated that age, sex, taking prescribed medication which affects heart rate, body mass index, and baseline PAI score were not associated with the change in PAI earned/day nor change in Total PAI achieved. However, for every month further from participants’ cardiac event, the change in Total PAI decreased regardless of blinding PAI scores. This concurs with research by Claes et al. [8] who found that two years after outpatient CR, a decline in steps and minutes of physical activity was observed suggesting the further from a cardiac event, the less exercise is performed.

Our findings indicated that the change in PAI scores was not influenced by participants’ perception of ease of use of device, whether they used the vibration and colour features on the device, found the wearing of the device comfortable, the level of understanding of the PAI concept or by the number of exercise barriers identified. Similarly, the perception of participants regarding whether they would continue to wear the device was not influenced by the amount of change in PAI achieved. As this is the first study, to the authors’ knowledge to investigate PAI in the cardiac population, there is no literature to compare these results with.

EQ-5D-5L total scores and EQVAS scores were significantly different between the start and end of the trial. These results cannot be interpreted as being influenced by PAI as a further survey was not completed at 3 weeks when data became un-blinded. However, our results indicate that by enrolling and completing the trial participants significantly improved overall quality of life.

The registering and charging were generally viewed as being straightforward, however the syncing was more problematic. Previous literature have reported wearables that are perceived to be easy to use were more likely to be utilised [27]. Wang et al. [50] reported factors such as performance expectancy and effort required were main contributing factors in consumer acceptance of healthcare wearable devices. As syncing was reported as requiring more effort than expected, this requires improvement to ensure long-term feasibility of continuing to use the PAI. With advances in technology and with the addition of PAI into more WPAM, it is expected that these technical difficulties will be improved.

With respect to useability, participants that used the features including colour change, vibration and workout mode found these useful and the PAI score was of interest to many participants. According to Nelson et al. [38] this gamification and interest may improve health empowerment in smart wristband users, however these authors also identified attractiveness as being important. Our interview findings supported this as a couple of female participants identified lack of attractiveness of the WPAM as a deterrent to long term use.

The results of our thematic framework identified participants were dissatisfied with some aspect of the comfort of the device. This may contribute to non-adherence long term and a couple of participants developed skin irritation. Buenaflor and Kim [6] identified physical comfort and safety an essential consideration in participant acceptability of a device with harm being a significant barrier to long term acceptance.

Having participants autonomously monitoring PAI resulted in increased motivation to exercise, and no participants reported the use of the approach demotivating. It is encouraging to see that the percentage of days where Total PAI and PAI earned/day between 25 and 50 PAI/week significantly increased in people with cardiac disease whose blinded score was less than 50 PAI/week. For participants already achieving 100 PAI/week in the blinded period, monitoring PAI resulted in participants remaining above 100 PAI, with half of these reaching 200 PAI and above. A previous study by Kieffer et al. [25], has shown that keeping PAI above 100 PAI/week maximises health benefits however there is no data on whether a score may be too high and result in harm. This knowledge would be particularly important for people with cardiac disease. One participant performed less PAI in the un-blinded period as he was already doing well above 100 PAI and as this higher level is unknown, he reduced his activity.

A systematic review by French et al. [15] reported goal setting, if specific and related to desired behaviours in older adults, significantly increased physical activity and resulted in greater motivation to be physically active and a qualitative analysis by Floegel et al. [14] reported self-regulation behaviours may enhance physical activity. Monitoring PAI creates self-regulation of physical activity and provides an avenue for goal setting which may therefore positively influence long term use. French et al. [15] also showed that heart rate monitoring, as a physiological feedback mechanism allowed increased self-efficacy when exercise was performed with no adverse effects. PAI may therefore improve self-efficacy through similar mechanisms.

Of those participants who had previously utilised a different WPAM our results showed most preferred the Lynk2 device as it was perceived as more accurate, and participants found the PAI concept of interest. Those that preferred other devices stated increased functionality such as displaying time, phone calls and texts outweighed the WPAM. This is in line with previous research that showed smartwatch usage is influenced by perceived usefulness more so than ease of use [7].

At the end of the trial period, many participants indicated they were intending to continue to wear the smartwatch and monitor PAI or were still considering continuation. This is line with the pilot randomised PAI e-health program used for people with type 2 diabetes which reported 80% of participants intended to continue PAI monitoring [9]. Participants stated they would recommend the device to others based on their interest in the PAI concept and its ability to monitor their health. Device attractiveness has been found to be important for some people for long term usage of wearable devices and a couple of our female participants mentioned this would have an impact on their decision to use the WPAM long term. This requirement is further supported by Choi and Kim [7] who found the ability of a device to allow for unique self-expression through choices in fashion such as changeable watchbands and colours were imperative for some consumers to continue to use smartwatches long term.

There has been ample literature reporting on the barriers to exercise identified by cardiac patients. A literature review by Santaularia and Jaarsma [47] identified logistical problems, lack of motivation to exercise, lack of time to exercise, laziness and inadequate social support as key factors influencing adherence to exercise in cardiac patients. Our participants identified similar barriers, except for inadequate social support. Additionally, our participants also found environmental factors and health status were key influencers in engaging in exercise. Health status was also found to be a barrier for exercise in cardiac patients in a review by Petter et al. [41]. Our findings also identified similar barriers to that found in a systematic review for non-completion of high intensity interval training exercise research trials (lack of interest and motivation, other commitments and medical issues) [19].

### Strengths of the study

Using a concurrent mixed methods approach, described in detail to improve dependability, allowed us to quantify the effect PAI monitoring via WPAM had on the amount and/or intensity of exercise. This method also allowed us to explore participants’ perceptions of ease of use, preferred functionality and comfort of the device; concept of PAI, future intention to use and likelihood of recommending the device and PAI to others. This resulted in a richer representation of participant experiences and views of the usefulness of PAI for people with cardiac disease and improved trustworthiness of the data. This methodological framework assisted in answering the breadth of our research questions, improving the quality of the trial.

To improve the rigour and credibility of our findings, a second researcher was tasked to agree on themes for our thematic framework analysis. Authors have different professional backgrounds further adding confirmability to our findings by implementing peer review from different perspectives. Vigour was improved by our use of verbatim quotations.

### Limitations of the study

The trial had several methodological limitations. The sample size was small and may have been underpowered. Caution should be taken when interpreting definitive findings from this exploratory trial. Females were underrepresented. The small sample size may have underestimated the contribution of factors analysed in the linear regression analyses. As this was the first study to use PAI as an intervention, a power calculation was not used.

The exercise period extended across the Christmas/New Year period with competing responsibilities which appeared to have negatively influenced exercise habits. This, along with the heat of summer, reduced the ability of participants to exercise and may have underestimated the potential impact PAI monitoring may have within the cardiac population. In addition, there may have been potential bias due to variability of contact time with participants whereby those with technical issues and device faults were assisted more than other participants, however, the researchers were careful not to discuss the trial during this time. Researchers only engaged in resolving technical issues.

The trial period was of short duration and the first week of the three was needed to generate a weekly Total PAI score, leaving two weeks to average the daily Total PAI score. Due to the short duration of data collection, results cannot be transferable to long term exercise. Although this was an exploratory study, our results are promising and found that PAI monitoring significantly increased exercise amount and/or intensity and most participants planned to continue to monitor PAI after completion of the trial. A cross over study design could have led to more conclusive findings.

### Future studies

The results of our study indicate a larger sample size, with longer duration monitoring, are also recommended to support our findings that PAI monitoring is of value and assists people with cardiac disease to increase the amount and/or intensity of physical activity. Longer duration studies could also investigate the effect of PAI monitoring on mortality in this population.

Having a more even distribution of sex of participants would also be beneficial to learn more about the effect of sex on PAI monitoring and WPAM. Consideration of time of year, spanning across seasons, may be beneficial to further explore barriers to exercise. Further collection of data about other barriers such as social support, income level, and occupational status would also be advantageous as these have been identified as barriers to exercise in the cardiac population in previous literature.

There were some participants who did not fully understand how to gain PAI and consideration of introducing individual sessions to further educate by comparing different activities and intensities in real time may assist in future studies.

Finally, future studies which use PAI monitoring on a device with improved syncing processes, increased functionality and attractiveness is also recommended to improve the likelihood of longer-term use.

## Conclusion

This exploratory study showed monitoring PAI via a WPAM increased the amount and/or intensity of physical activity within the cardiac population. Participants found the concept of PAI interesting, beneficial, and motivating. If WPAM syncing and aesthetics improved, along with offering greater functionality in line with comparative smart devices, participants may continue to use the device long term. Participants would recommend monitoring PAI to others, particularly due to the individual calculation which is affected by intensity of exercise. PAI may be a viable strategy to assist people with cardiac disease maintain physical activity adherence.

## Supplementary Information

**Additional file 1:** Qualitative Questionnaire for Semi-Structured interviews. A questionnaire developed by authors to help facilitate semi-structured interviews.

**Additional file 2:** COREQ Checklist. The Consolidated Criteria for Reporting Qualitative Studies Checklist. A 32-item checklist for reporting qualitative data

## Data Availability

The datasets generated and/or analysed during the current study are available https://research.bond.edu.au/en/datasets/effect-of-physical-activity-intelligence-pai-monitoring-in-the-ma. https://doi.org/10.26139/GTYS-6M55.

## Abbreviations

PAI: Personal activity intelligence
WPAM: Wearable physical activity monitoring device
CVD: Cardiovascular disease
CHD: Coronary heart disease
CR: Cardiac rehabilitation
CRF: Cardiorespiratory fitness
EQ-5D-5L: Euro quality of life -5 dimension-5 level health questionnaire: English version for Australia
