# Supplementary material for: Effect of personal activity intelligence (PAI) monitoring in the maintenance phase of cardiac rehabilitation: a mixed methods evaluation
Source: BMC Sports Sci Med Rehabil. 2021 Oct 10;13:124. doi: 10.1186/s13102-021-00350-9 (PMC8503999; doi:10.1186/s13102-021-00350-9)
Supplement: Supplementary file 1 — Additional file 1: Qualitative Questionnaire for Semi-Structured interviews. A questionnaire developed by authors to help facilitate semi-structured interviews. [file 13102_2021_350_MOESM1_ESM.docx]

**Supplementary File 1:**

**Qualitative Questionnaire for Semi-structured Interviews**

| Question | Easy/ | |  | |  | Difficult |
| --- | --- | --- | --- | --- | --- | --- |
| Please rate by ticking the most appropriate box in answer to the following questions: | 1 | | 2 | 3 | 4 | 5 |
| Ease of registering |  | |  |  |  |  |
| Ease of syncing the Lynk 2 device |  | |  |  |  |  |
| Ease of Charging the Lynk 2 device |  | |  |  |  |  |
| Ease of filling out the exercise diary |  | |  |  |  |  |
| Please rate how difficult you found not being able to look at the Lynk 2 device during the first half of the study? |  | |  |  |  |  |
| How likely are you to continue wearing the Lynk 2 device? | | Not likely |  |  |  | Highly likely |
| Please rate the degree of change you felt in motivation to exercise after receiving education about PAI? | | No change |  |  |  | Considerable change |

1. Could you please expand on the reasons for your ratings in the questionnaire?
2. Could you describe your thoughts about wearing the LYNK 2 device?
3. Were there any obstacles to wearing the Lynk 2 device and if so, what were they?
4. Please describe how you felt about not being able to view the data in the first half of the study?
5. Generally, what barriers, if any, do you have to exercising regularly?
6. Were you sick during the trial?
7. Did the week of rain/smoke/heat influence the amount of exercise you did or did not do?
8. Did the information regarding PAI interest you and if so, in what way? If not, why?
9. How did having this information, plus being able to see the Lynk 2 device, influence your exercise habits?
10. In what way did the Lynk 2 device influence the amount of exercise you performed?
11. What did you enjoy, and not enjoy, about having a Lynk 2 device to help monitor your activity?
12. Which features of the Lynk 2 device did you find most useful and why (vibration, change of colour etc)?
13. Would you, if given the option, continue to use the Lynk 2 device or not and what made you come to this decision? If no, was this because of the device or the PAI metric? Would you be interested in continuing with PAI monitoring if it was on another device?
14. Do you own any other fitness trackers? If so, which device (your own or Link 2 device) do you prefer? Why? Which device do you think you are more likely to utilise? Why?
15. What additional strategies do you believe may be required to assist you to increase your activity habits? Ie daily texts (would this help or hinder?
16. Would you recommend this device to others? Why or Why not?
17. How did you feel about exercising at home once you finished cardiac rehabilitation?
18. Do you believe you were given adequate guidelines around exercise after the outpatient cardiac rehabilitation programme?
